# A Genome-Wide Survey of Switchgrass Genome Structure and Organization

**DOI:** 10.1371/journal.pone.0033892

**Published:** 2012-04-12

**Authors:** Manoj K. Sharma, Rita Sharma, Peijian Cao, Jerry Jenkins, Laura E. Bartley, Morgan Qualls, Jane Grimwood, Jeremy Schmutz, Daniel Rokhsar, Pamela C. Ronald

**Affiliations:** 1 Department of Plant Pathology, University of California Davis, Davis, California, United States of America; 2 Joint BioEnergy Institute, Emeryville, California, United States of America; 3 China Tobacco Gene Research Center, Zhengzhou Tobacco Research Institute, Zhengzhou, China; 4 HudsonAlpha Institute of Biotechnology, Huntsville, Alabama, United States of America; 5 United States Department of Energy Joint Genome Institute, Walnut Creek, California, United States of America; 6 University of California, Berkeley, California, United States of America; USDA-ARS, United States of America

## Abstract

The perennial grass, switchgrass (*Panicum virgatum* L.), is a promising bioenergy crop and the target of whole genome sequencing. We constructed two bacterial artificial chromosome (BAC) libraries from the AP13 clone of switchgrass to gain insight into the genome structure and organization, initiate functional and comparative genomic studies, and assist with genome assembly. Together representing 16 haploid genome equivalents of switchgrass, each library comprises 101,376 clones with average insert sizes of 144 (*Hin*dIII-generated) and 110 kb (*Bst*YI-generated). A total of 330,297 high quality BAC-end sequences (BES) were generated, accounting for 263.2 Mbp (16.4%) of the switchgrass genome. Analysis of the BES identified 279,099 known repetitive elements, >50,000 SSRs, and 2,528 novel repeat elements, named switchgrass repetitive elements (SREs). Comparative mapping of 47 full-length BAC sequences and 330K BES revealed high levels of synteny with the grass genomes sorghum, rice, maize, and *Brachypodium*. Our data indicate that the sorghum genome has retained larger microsyntenous regions with switchgrass besides high gene order conservation with rice. The resources generated in this effort will be useful for a broad range of applications.

## Introduction

The C4 perennial grass, switchgrass (*Panicum virgatum* L.), a member of Paniceae tribe of the Panicoideae subfamily of the Poaceae is a promising bioenergy crop [1], [2]. Striking features include its high productivity, adaptability to growth on marginal lands, low nutrient and water requirements, and ability to sequester carbon and recycle nutrients [3], [4], [5], [6], [7].

The work reported here is part of an effort directed towards generating the genetic and genomic resources for switchgrass needed for gene discovery and breeding efforts [8], [9]. Considering the highly outcrossing and tetraploid features of lowland switchgrass with two heterozygous genomes [10], major challenges will be independently assembling the subgenomes into a reference and reaching chromosome-scale contiguity. An accurate estimate of genome structure and composition prior to full genome sequencing is needed. Generation and sequencing of BAC libraries is an efficient strategy to obtain this information and support assembly of the large and complex underlying genomes [11], [12], [13], [14], [15], [16]. Recently, an *Eco*RI-generated BAC library was reported from the SL93 2001-1 genotype of Alamo switchgrass [17]. Based on the analysis of homoeologous genomic regions harboring orthologs of the rice *Brassinosteroid insensitive 1* (*OsBRI1*), those authors made an attempt to provide a glimpse of switchgrass genome structure and complexity. However, the analysis was limited to a single locus and only one restriction enzyme (*Eco*RI) was used. Additional libraries are required to achieve unbiased and near-complete representation for genome-wide studies.

Here, we describe the generation and characterization of two high-quality BAC libraries using two different restriction endonucleases (*Bst*YI and *Hin*dIII) prepared from the switchgrass genotype Alamo clone AP13. Because this clone was the parent of the first mapping population described for switchgrass and has been further used in defined crosses [18], it was chosen as the consensus target for sequencing by the switchgrass community. Collection of 330,297 high-quality BAC-end sequences (BES) were generated from both the libraries that provided the basis for a genome-wide survey of switchgrass genome structure and organization. Comparative mapping of full-length BACs and BES onto four other grass genomes reveals high levels of synteny and micro-collinearity. Gene annotations and analysis of BES provide an estimate of protein signatures, GC content, repeat elements and SSRs in switchgrass genome.

## Results

### Construction and Characterization of BAC Libraries

**Figure 1:**
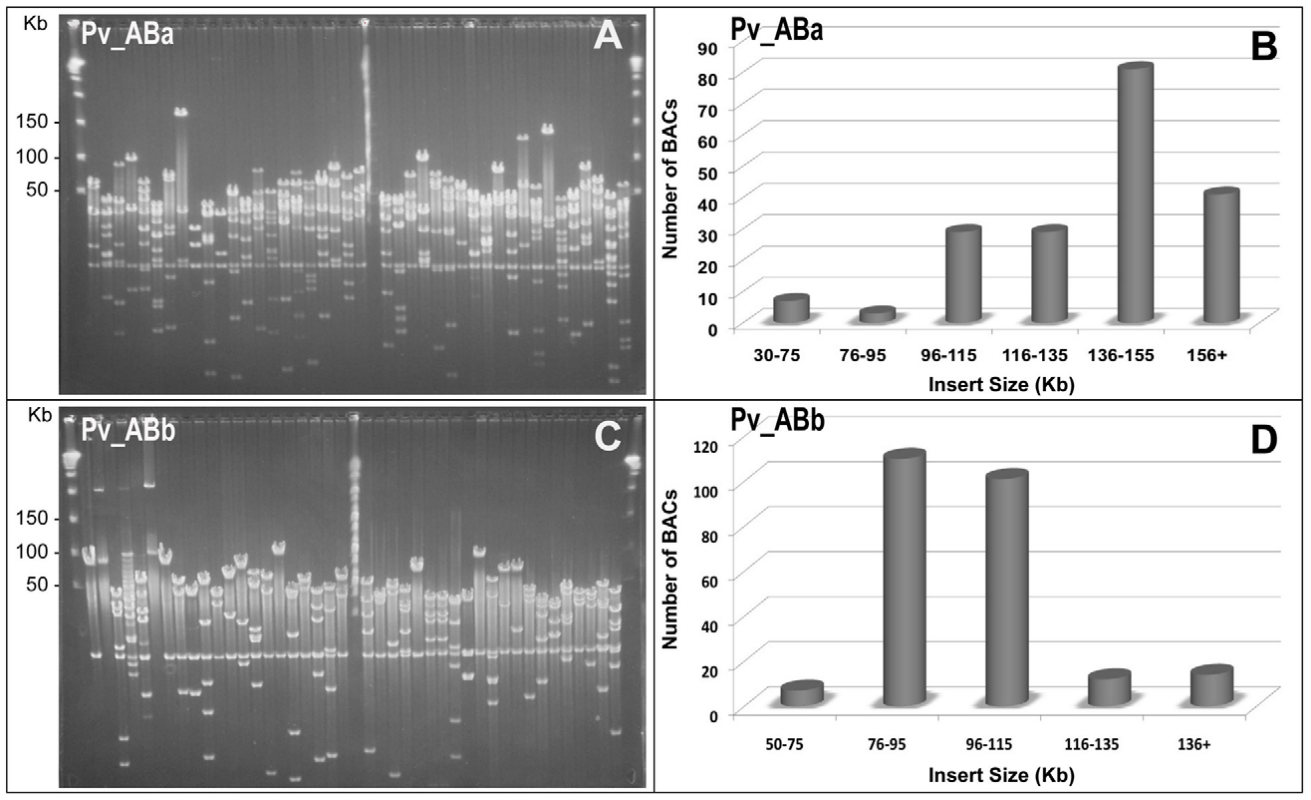
Determination and distribution of switchgrass BAC clone insert sizes. DNA was digested from >180 randomly selected BAC clones from Pv_ABa and Pv_ABb libraries and analyzed by Pulsed Field Gel Electrophoresis. A, C), Representative gel pictures of *Not*I digested BAC DNA from Pv_ABa and Pv_ABb libraries, respectively. B, D) Estimated BAC insert sizes with their relative frequencies.

We constructed two BAC libraries, Pv_ABa and Pv_ABb, from AP13 clone of switchgrass using *Hin*dIII and *Bst*YI, respectively. Each library consists of 101,376 clones. To estimate insert size, >180 clones were randomly picked from each library. *Not*I digestion of these clones generated 7.8 kb vector band and various-sized insert fragments (Figure 1A, C). The inserts in Pv_ABa ranged from 30 to 280 kb, with the majority of fragments in the 136–155 kb size range (Figure 1B) and an average size of 144 kb. For Pv_ABb, insert sizes ranged from 50 to 200 kb, with the majority of fragments in the 76–115 kb size range and an average size of 110 kb (Fig. 1D). More than 80% of tested clones from both libraries had an insert size larger than 100 kb. A very low percentage (<1%) of empty clones were detected in both the libraries. The detailed characteristics of both the libraries are summarized in Table 1.

**Table 1:** Characteristics of the switchgrass BAC libraries.

Characteristic	Pv_ABa	PV_ABb
Cloning vector used	plindigoBAC536	plindigoBAC536
Restriction enzyme	*Hin*d III	*Bst*Y I
Total number of clones	101,376	101,376
Percent empty clones	<1%	<1%
Maximum insert size	280 Kb	200 Kb
Minimum insert size	30 Kb	50 Kb
Average insert size	144 Kb	110 Kb
Chloroplast DNA contamination	0.57%	0.17%
Mitochondrial DNA contamination	0.79%	0.29%
Number of genome equivalents	9X	7X

**Figure 2:**
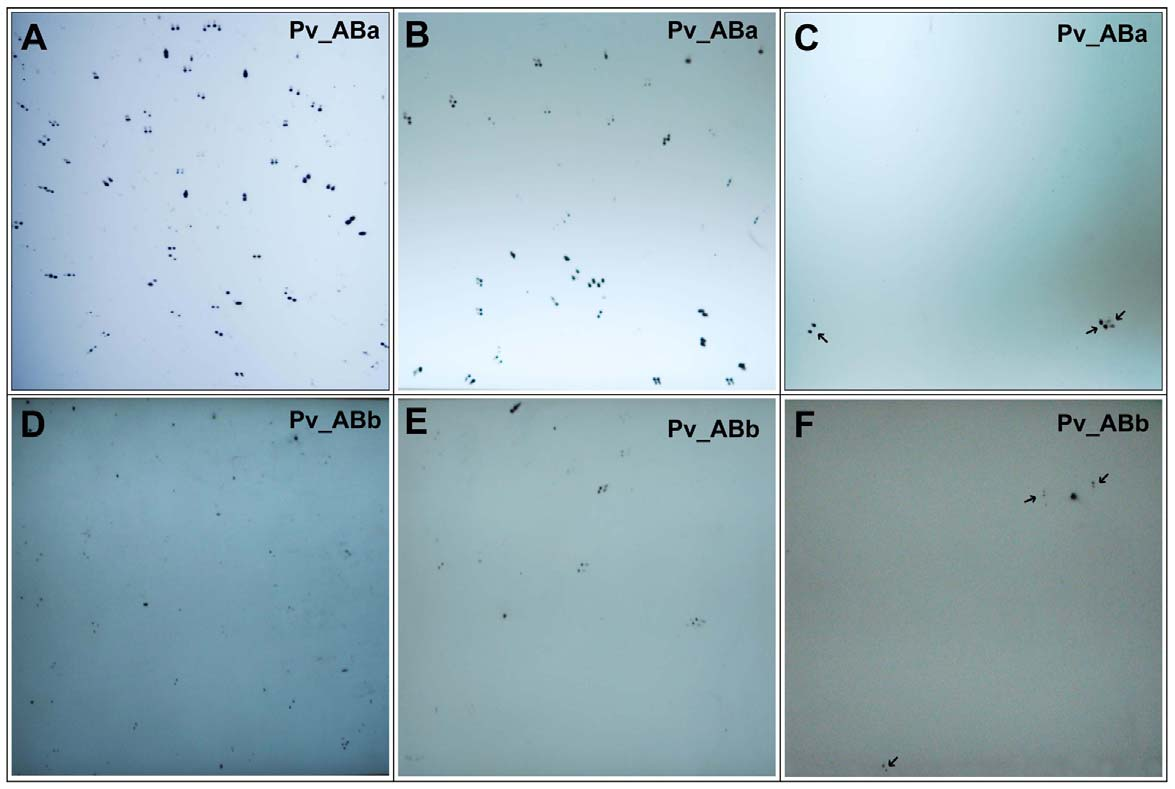
Estimation of organellar DNA contamination and representation of low-copy genes. Switchgrass BAC libraries (Pv_ABa and Pv_ABb) were screened by high-density filter hybridizations to estimate chloroplast- or mitochondrial-specific DNA and representation of single/low copy genes. Representative filter hybridization data used to estimate chloroplast (A, D) and mitochondrial (B, E) contaminants, and the library coverage based on the presence of a single copy gene, *brittle culm 10* (C, F). Black arrows in C and F identify the signal from *BC10* probes.

To assess the quality of the BAC libraries, high-density colony filters were hybridized with chloroplast/mitochondria-specific probes spanning the whole genome of respective organelle. Using a pool of chloroplast-specific genes, viz., *rbc*L, *ndh*A, *rpo*B and *trn*L, 209 and 62 clones among 36,864 clones from Pv_ABa and Pv_ABb BAC libraries, respectively, produced hybridization signal. We, therefore, estimate that 0.57 and 0.17% clones in Pv_ABa (Figure 2A) and Pv_ABb (Figure 2D), respectively, carry chloroplast-originated DNA sequences. Similarly, hybridizations with the mitochondrial DNA probe containing mixture of *atp*6, *atp*9, *cob* and *cox*1 gene-specific amplicons identified 79 and 23 mitochondrial clones in Pv_ABa (Figure 2B) and Pv_ABb (Figure 2E), respectively. This amounts to 0.21 and 0.06% contamination from mitochondrial clones in Pv_ABa and Pv_ABb library, respectively. The overall contamination of organellar DNA in Pv_ABa and Pv_ABb is, therefore, estimated to be 0.78 and 0.23%, respectively.

### Coverage of the Switchgrass Genome

Prior analyses suggest that switchgrass is an allotetraploid with an effective genome size of 2x = 1n = 1600 Mbp [19]. Considering the ∼1600 Mbp effective genome size of *Panicum virgatum* L. var. Alamo and removing estimated organellar DNA-specific (0.78 and 0.23%) as well as empty clones (1%), each library represents ∼9 and 7 haploid genome equivalents. Therefore, the theoretical probability of finding a sequence of interest in these library resources is more than 99.9%.

We empirically validated the coverage using filter hybridizations with single/low copy genes (Figure 2C, F). The copy number of six genes, including *brittle culm 10* (*BC10*), *xyloglucan endotransglucosylase/hydrolase* (*OsXTH*) and, *Teosinte branched 1* (*TB1*) of rice and *Tubulin-4, Opaque,* and *Starch branching enzyme 1 (SBE1)* of maize, was determined using Southern hybridizations. In switchgrass, *OsXTH* and *TB1* appear to have several copies or exhibit variability among homoeologous regions, whereas, *BC10, Tubulin-4, Opaque* and *SBE1* have single or low copy number (Figure S1). Using a *BC10* gene-specific probe, three clones were identified among 18,432 clones of each library (Figure 2C, F). Similarly 3, 2 and 2 clones specific to *Tubulin-4, SBE1* and *Opaque*, respectively, were identified among 18,432 clones of Pv_ABa library (data not shown). Conversely, 2, 1 and 3 clones were identified for *Tubulin-4*, *Opaque* and *SBE1*, respectively, from the second library. Therefore, an average of two clones were obtained per single/low copy gene in the 18% of the clones represented on the filters, corresponding to about 11 hits in each library and consistent with the high coverage of each BAC library.

### BAC-end Sequencing and Analysis

**Figure 3:**
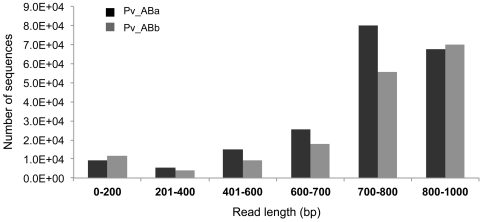
Size distribution of BAC-end sequences. The x-axis represents read length with the numbers of sequences indicated on y-axis.

Because BES data represent a random snapshot of a genome, it can be used to perform a genome-wide survey of structural features. We sequenced paired ends of 101,376 and 84,480 clones from Pv_ABa and Pv_ABb, respectively. After removing *E. coli*-specific sequences, vector sequences, short/failed sequences, and organelle-specific DNA, a data set of 330,297 (∼263 Mbp) high quality sequences (> = 400 HQ bases) was generated. These represent ∼16.4% of the switchgrass genome. 95.9% BES were paired. The length of BAC-end sequences varied from ∼100 to 1000 bp with an average length of 761 bp (Figure 3). More than 73% clones of each library had a read length longer than 700 bp. Based upon homology with coding sequences from other grass genomes and the presence of protein domains, approximately, 15.4% (40 Mbp) of BES had a protein signature. A protein signature refers to the contiguous pattern of amino acids associated with a particular structure or function of proteins [20]. Based on the BES analyzed, the GC content of switchgrass is estimated to be ∼45.5%. Further, GC content in the sequences with a protein signature is 57.8%, which is significantly higher that the GC content (43.3%) of non-coding region in the BES (222 Mbp).

#### Analysis of Simple Sequence Repeats

**Figure 4:**
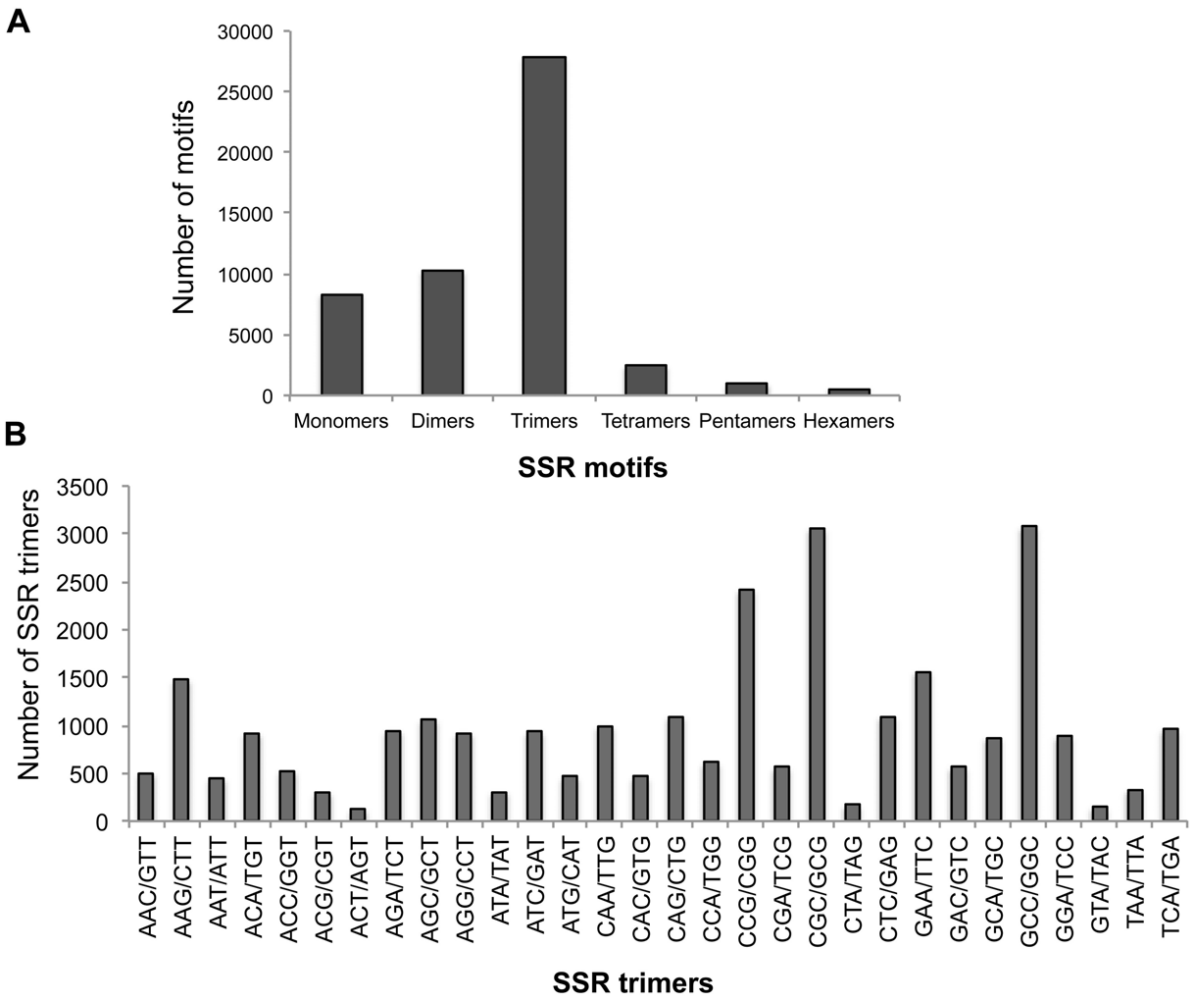
Analysis of Simple Sequence Repeats (SSRs) from BAC-end sequences. **a**) Distribution of total number of repeat loci. The x-axis represents the length of SSRs and the y-axis indicates total number of motifs observed. **b**) Distribution of SSR trimers. X-axis represents the various trinucleotide SSRs and the y-axis represents number of SSRs.

We identified a total of 50,206 SSRs from BES that includes 1–3 nt repeats (at least 12 nt in length) and 4–6 nt repeats (having at least four tandem repeat units) adding up to 870,808 bases. The density of SSRs is therefore, estimated to be one SSR per 5.2 kb of sequence. The most abundant of these were trimeric SSRs (55%,), followed by dimers (20.4%) and monomers (16.6%; Figure 4a). However, tetramers, pentamers and hexamers were much lower in abundance and all together add up to less than 10% of total microsatellites. Furthermore, GC-rich trimers constitute 63% of total trimers with GCC/GGC and CGC/GCG being most abundant (Figure 4b). ACT/AGT trinucleotides were least in number (Figure 4b). About 14% of the SSRs (6812 in number) were longer than 20 nucleotides. Details of SSRs and their frequencies are given in File S1.

#### Analysis of Repetitive Elements

Based upon homology with known plant repeat elements, 279,099 repeat elements were identified from the switchgrass BES (Table 2). Such repeats correspond to 30.97% of the total sequence analyzed. Class I and class II transposons account for 73.7 and 26.3% of total transposons, respectively, thereby suggesting an approximate ratio of 3∶1 in the switchgrass genome. Class I transposons include Long Terminal Repeats (LTR) elements, Short Interspersed Elements (SINEs), and Long Interspersed Elements (LINEs). LTR-elements were most abundant and comprise 90.4% of total retrotransposons identified; however, SINEs (1.2%) and LINEs (8.3%) were very low in number (Table 2). LTR-elements are further classified into five major groups including BEL, Ty1/Copia, Ty3/gypsy, DIRS1 and vertebrate retroviruses. We did not find any BEL or retrovirus type elements in switchgrass. Ty3/Gypsy and DIRS1 together comprise 67% of the LTRs in switchgrass. Similarly, Class II transposons include 35% En-spm, 13.5% Tourist/Harbinger, 17.8% MuDR-IS905, 12.4% Hobo-Activator, 9.06% Tc1-IS630-Pogo and others (8.7%). Based on these results, we estimate that ∼31% of the switchgrass genome corresponds to known repeat sequences. Several retro-element subfamilies including Penelope, CRE/SLACS, L2/CR1/Rex, R1/LOA/Jocky, R2/R4/NeSL, BEL/Pao, Rolling-circles and, DNA transposons viz., PiggyBac, Mirage, P-element and Tarnsib were not found in the sequence analyzed.

**Table 2:** Distributions of repeat elements identified from switchgrass BAC-end sequences.

Type of Element	Total number of elements*	Total length occupied (bp)	%age of total sequence analyzed
**Retroelements**	178318	64563658	24.53%
SINEs	2306	348493	0.13%
Penelope	27	3174	0.00%
LINEs	14816	6018230	2.29%
R2/R4/NeSL	2	106	0.00%
RTE/Bov-B	3780	1911841	0.73%
L1/CIN4	11001	4102823	1.56%
LTR elements	161196	58196935	22.11%
Ty1/Copia	50870	18995037	7.22%
Ty3/Gypsy/DIRS1	108785	38941535	14.79%
**DNA transposons**	63616	14149503	5.38%
hobo-Activator	7917	1872711	0.71%
Tc1-IS630-Pogo	5764	867375	0.33%
En-Spm	22287	6169405	2.34%
MuDR-IS905	11351	2572080	0.98%
Tourist/Harbinger	8620	1441495	0.55%
Others	-	1226437	0.47%
**Unclassified**	4424	804772	0.31%
Total interspersed repeats:		79517933	30.21%
Small RNA:	2382	568637	0.22%
Satellites:	1634	244518	0.09%
Low complexity:	28725	1199167	0.46%

* most repeats fragmented by insertions or deletions have been counted as one element.

#### Identification of Novel Repeats

**Figure 5:**
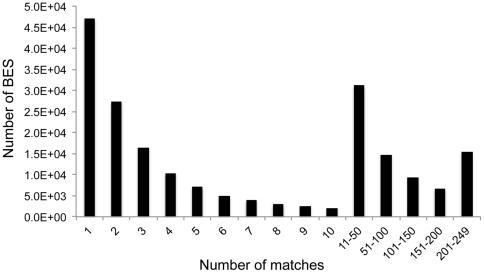
Distribution of BAC end sequences that show significant homology to other BES. The x-axis represents the number of matches and y-axis contains total number of BAC-end sequences.

Similarity-based repeat detection is generally limited by the size and diversity of the available databases. To identify switchgrass-specific novel repeat elements, we carried out a self-comparison of the BES. Even with the stringent threshold requirement that each 100 bp window matches another BES with at least 90% identity, 61.2% (202,280) of the switchgrass BES matched at least one other BES (Figure 5). We identified 2,948 repeat sequences among those BES with at least six matches with other switchgrass BES. When these sequences were queried against the RepBase repeat database, MSU Plant Repeat Databases, Triticeae repetitive sequence database (TREP), NCBI GenBank non-redundant nucleic acid sequence database and Swissprot database (release 2011_08), 420 repeat sequences matched at least one record in the mentioned databases and were therefore, removed from the list of putative switchgrass repetitive elements (SREs). The remaining 2,528 SREs were present in 7 to 548 copies in the BES database and their sizes ranged from 80 to 300 bp (File S2). Overall, these SREs matched 83,289 BES, covering a ∼6 Mbp region that accounts for ∼2.3% of the total BES length. Extrapolating to the level of the switchgrass genome, there could be as many as 3,341 copies of the most frequent SREs.

#### Functional Annotation and Gene Ontology Analysis

**Figure 6:**
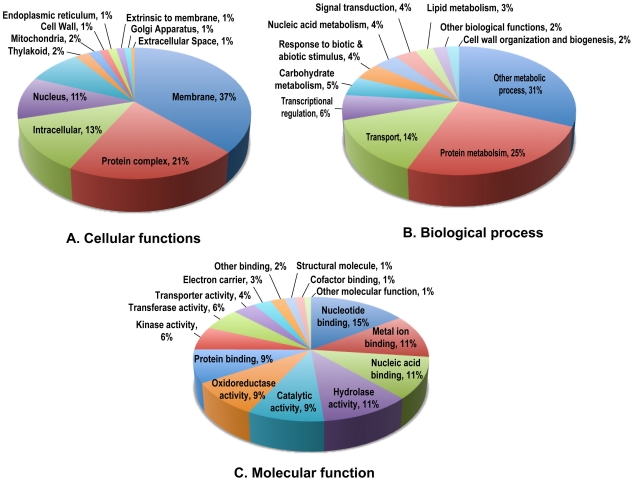
Distribution of GO-slim annotations of putative gene products predicted from switchgrass BAC-end sequences. A, Cellular locations −12 groups of gene ontology; B, Biological processes −11 groups of gene ontology; C, Molecular functions −15 groups of gene ontology terms.

To better characterize this valuable resource and provide an overview of the expanse of biological functions encoded by the switchgrass genome, we performed functional annotation and GO analysis of protein-coding signatures obtained from the BES with regard to the three major gene ontology terms viz., molecular function, biological process and cellular locations. Out of the 330,297 BES, 5052 could be associated with at least one GO term (File S3). In total, 716 terms were associated with 5052 reads. 4507 reads were assigned at least one of the 377 molecular function categories, 3244 reads were annotated with at least one of 259 biological function categories and 1144 reads were associated with at least one of the 80 cellular location categories. Figure 6 presents the distribution of GO terms identified from the switchgrass BES. The top most terms highlighted in the cellular location category included membrane (37%) and those comprising protein complexes (21%). Equal representation (11%) of those associated with nucleotide binding, metal ion binding, nucleic acid binding and hydrolase activity were found in the molecular function category followed closely by catalytic activity (9%), oxidoreductase activity (9%) and protein binding (9%) terms (Figure 6). With regard to biological functions, terms associated with metabolic processes were most abundant, followed by transporters (14%) and transcriptional regulators (6%). Overall, genes annotated to encode kinases, transcription factors, metal ion binding proteins and oxidoreductases comprise a large proportion of the coding regions of switchgrass genome.

#### Comparative Mapping of Switchgrass BES

**Figure 7:**
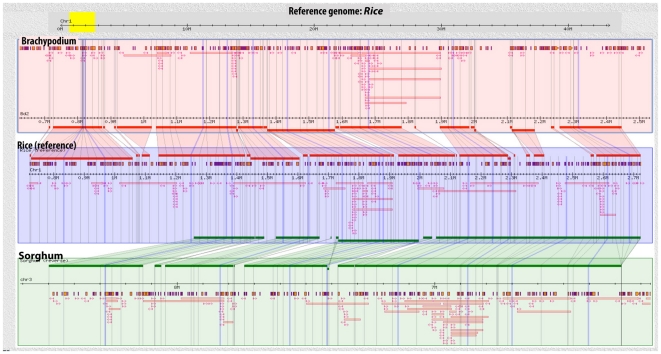
Mapping results of switchgrass BAC-end sequences to a 2 Mbp region of rice with orthologous regions from sorghum and *Brachypodium.* BES having base pair identity >75% with e value <1e-20 and coverage of >50% were placed on to rice peptides. The equivalent regions in sorghum and *Brachpyodium* were identified and used for mapping BES. BES pairs that were placed within 500 kb are represented by red bars. Red blocks show high-quality singlets of switchgrass with arrows indicating orientation. Synteny between grass genomes is marked by mauve alignment.

For comparative mapping, we initially mapped switchgrass BES to rice peptides, which were subsequently mapped onto sorghum and *Brachypodium* genomes. A GBrowse-based synteny browser, GBrowse-syn [21], was used to display the synteny between the rice, sorghum and *Brachypodium* genomes. Approximately 8% of the BES mapped to sorghum, 7% to rice, and 5.5% to the *Brachypodium* genome. In total, 4522 (1%) paired end reads mapped to sorghum; whereas, 24,758 (∼7%) reads mapped as high scoring singlets. Mapping onto the rice genome placed 2400 (0.7%) paired ends and 22,158 (6.4%) high scoring singlets. Similarly, 1568 (0.5%) paired ends and 17,517 (5%) high scoring singlets mapped onto the *Brachypodium* genome. Figure 7 displays a snapshot of a 2.0 Mbp region of rice with mapping results from corresponding regions of sorghum, *Brachypodium* and switchgrass BAC-end sequences. In the region, 332 BAC-ends mapped to sorghum, 298 to rice and 275 to *Brachypodium* genome. Forty-six BAC-end sequences that mapped to sorghum had both ends placed within 500 kb of one another. Similarly, 24 paired-BES were mapped to orthologous region in rice and 22 to *Brachypodium* genome. Based on the paired placements in the region shown in Figure 7; 74.7, 89.45 and 43.29% BES mapped to coding sequence in sorghum, *Brachypodium* and rice, respectively. The regions with both ends mapped within 500 kb represent microsyntenous regions in these genomes.

#### Analysis of Microcollinearity using Full-length BAC Sequences

Forty-seven randomly selected BACs from Pv_ABa were sequenced to essentially full-length using Sanger's method. The average size of these BACs was 153.6 kb. The distribution of SSRs and repeat elements in the full-length BAC sequences (File S4) is very similar to their distribution among the BES. A total of 439 gene loci (451 gene models; File S5) were annotated from ∼7.2 Mbp of switchgrass genomic sequence, obtained from full-length BAC sequences. The gene density is therefore estimated to be one per 16.4 kb of genomic sequence. Predicted cDNA, protein and genomic sequences of these loci are given in File S6. The genes predicted from these sequences were mapped onto other grass genomes. Corresponding orthologs for 370 (84%), 363 (83%), 357 (82%) and 336 (77%) gene loci could be identified from rice, maize, sorghum and *Brachypodium*, respectively (File S7).

**Figure 8:**
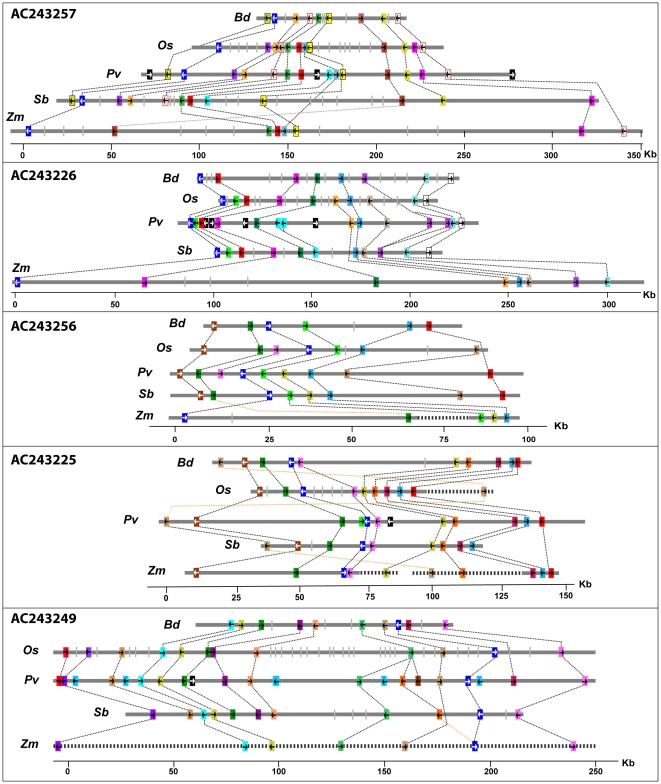
Micro-collinearity between switchgrass BAC clones and orthologous regions from *Brachypodium* (*Bd*), rice (*Os*), sorghum (*Sb*) and maize (*Zm*). Colored boxes along the physical location in the genome of each species represent genes and arrows in the colored boxes indicate the transcriptional orientation of each gene. Orthologous genes are given the same color and are connected by dotted lines. Grey bars represent genes from respective genomes lacking syntenic match in switchgrass. Dashed lines represent breaks in contiguity to allow larger genomic regions of the chromosomes to fit in the scale of the figures and the genes from these regions lacking syntenic match have not been plotted. The scale is shown at the bottom of each section. NCBI accession numbers for switchgrass BAC clones are given at top left of each section. Detailed information on accession numbers and gene names is given in File S7.

We compared the order of switchgrass genes and their transcriptional orientations with orthologous regions in sorghum, maize, rice and *Brachypodium*. Figure 8 shows the pictorial representation of micro-collinearity among five BAC clones of switchgrass and the corresponding regions in other sequenced grasses. Generally, the length of corresponding regions is longer in maize and smaller in *Brachypodium,* in agreement with the whole genome size rankings. Despite various local rearrangements in these regions including inversions, translocations, deletions and insertions, we generally observed a high level of micro-collinearity in terms of gene content. A few genes have undergone tandem duplication in switchgrass resulting in paralogs. The list of genes from rice, sorghum and *Brachypodium*, not represented in switchgrass, is given in File S8. Out of 47 BACs analyzed, half are significantly collinear with other grass genomes; whereas, the rest show varying rearrangements (File S7). Reduced collinearity in some of the BACs seems to be due to low representation of coding sequences in these BACs. Overall, order, transcriptional orientations and gene structures of switchgrass genes seem more conserved with those of rice and sorghum, than those of maize and *Brachypodium* (Figure 8).

## Discussion

### High Quality BAC Libraries Provide a Valuable Resource for Diverse Genetic and Genomic Studies in Switchgrass

While trying to assemble the tetraploid genome of switchgrass, a major challenge will be to discriminate between paralogous, orthologous and homoeologous regions. Further repetitive regions longer than the read length and similarity in homoeologous regions may lead to potential misassemblies, which could require a great deal of directed sequencing to accurately resolve [22]. An ordered clone sequencing [23] approach using large insert clones can assist in assembly of the shorter genome sequences generated by next generation sequencing technologies [24], [25], [26]. BAC libraries are preferred over fosmid, cosmids or yeast artificial chromosomes, for this purpose because of their ability to preserve larger DNA fragments and lower level of chimerism [27], [28], [29], [30], [31].

Here we report construction of two BAC libraries from switchgrass accounting for ∼16 haploid genome equivalents of switchgrass with >99.9% probability of finding a particular sequence. The large insert size, high coverage and low organellar DNA contamination indicate that these libraries provide a useful resource for diverse genetic and genomic studies including genetic and physical mapping, exon trapping, isolation of closely-linked polymorphic markers, FISH analysis, as well as functional and comparative genomics studies [28], [32], [33], [34], [35]. The percentage of empty clones observed (∼1%) is also comparable or significantly lower than other reports for maize (0.4%; [36]), *Panax ginseng* (2.7%; [37]), *Vitis vinifera* (2.2%; [38]) and *Brachypodium* (4.6 and 5.1%; [39]). As these libraries have been constructed from the same clone (AP13) that is being sequenced at JGI, the sequences generated will prove instrumental for assembly and gap filling of the genome sequence of switchgrass.

### GC-rich Trinucleotides are the Most Abundant SSRs in Switchgrass

Microsatellites play an important role in genome evolution and gene regulation. They have been extensively used in several research areas including linkage mapping, comparative genomics and population genetics [40], [41]. Monocot genomes are enriched in GC-rich SSRs [42] with trinucleotide SSRs being most abundant in sorghum, maize and rice genomes (File S9; [43]. We find that switchgrass also, trinucleotide SSRs predominate (55.3%), with 63% of them being GC-rich, reflecting the codon bias. These observations are similar to the results observed for rice (65%) and *Brachypodium* (67.4%). Distributions of SSRs in full-length BAC sequences also showed similar distribution patterns as identified with BES. In plants, a negative correlation exists among SSR density and genome size [42] and our data also conforms to this general trend (File S9). Out of >50,000 SSR sequences discovered here, 6,812 are longer than 20 nucleotide in length and will serve as a valuable resource to develop highly heterozygous and polymorphic markers for saturating existing linkage maps.

### Repeat Content in Switchgrass is Estimated to be ∼33%

Transposable elements are abundant in plant genomes and play an important role in determining the size of grass genomes and driving genome evolution in response to environmental cues [44], [45]. Known repeat elements accounted for approximately 31% of the total BES analyzed, with transposable elements representing about 86.7% of the repetitive-DNA fractions. Therefore, the estimated transposon content in switchgrass is approximately 29.9%. The percentage of retroelements in switchgrass (24.53%) is more than double compared to *Arabidopsis* (10%; [46]), similar to that of rice (26%), half of sorghum (55%) but less than one third of maize (79%; [47]). Analysis of full-length BAC sequences also showed similar patterns (File S4). Similar to poplar, rice and sorghum [48], the Gypsy group of LTRs is the most abundant repetitive elements in switchgrass. The ratio of Gypsy to Copia elements in switchgrass is ∼2∶1, similar to the ratio reported for rice [49]. LTRs have not only been implicated in genome reorganization but are also involved regulating plant adaptation to biotic and abiotic stresses [50]. Therefore, these elements might have significant contribution in stress adaptation and shaping the switchgrass genome.

In addition to the repetitive DNA fraction identified by classical analysis (30.97%), novel SREs (∼2.3%) bring the total repetitive DNA content of switchgrass to a minimum of ∼33% which is similar to estimated repeat content in rice in spite of the much greater genome size of switchgrass (File S9).

### GC Content in Switchgrass is Comparable to Other Grasses

GC content is an important feature of a genome as indicated in several studies of prokaryotes, vertebrates and plants [51], [52], [53], [54]. Gene density, patterns of codon usage, distribution of repeat elements, methylation patterns and recombination rate are all associated with GC content [52], [55], [56]. GC content is correlated with codon bias specifically at the third position and is reported higher in monocot plant species (File S9; [56]). Based on BES data, the estimated GC content in switchgrass is 45.5%, which is comparable to other monocot species (File S9). However, GC content of coding regions (57.8%) is noticeably higher than that of non-coding regions (43.3%), which may be the result of GC-rich codon usage and will be important for gene annotations of this species [57].

### Gene Density in Switchgrass is more similar to that of Rice

Due to its large genome size, the genes in switchgrass are expected to have longer intergenic regions as compared to rice and other shorter genomes. Based on BAC-end sequence analysis, the estimated gene density in switchgrass is one gene per 16.4 kb, which varies in gene-rich and gene poor or repetitive regions. The highest density observed among the full-length BAC sequences is one gene per 6.8 kb (AC243226) and lowest was one gene per 59.4 kb (AC243244). Conversely, gene density in rice, sorghum and *Brachypodium* is one gene per 13.4 Kb, 26.7 Kb and 10.6 Kb, respectively [58]. However, gene density in maize is estimated to be three times lower than that of rice [59]. Closer inspection of some BACs suggested that in the regions of high gene density, most of the genes are clustered within a short distance. Therefore, the gene arrangement in switchgrass is more similar to that of rice.

### Synteny and Collinearity of Switchgrass with Evolutionarily Diverged Grass Species

Investigation of genomic organization and comparative mapping to other grasses using RFLP (restriction fragment length polymorphism) markers revealed several syntenic regions between the rice and switchgrass genomes. [18]. Similarly, ESTs and other marker-based studies have also revealed significant similarity of switchgrass genome to sorghum, pearl millet and rice [10], [60], [61], [62]. However, conservation of marker order at the level of a genetic map may not reflect the micro-collinearity at the genic level [63], [64]. Sequence comparisons at various loci have shown that local rearrangements including deletions, insertions, duplications and translocations have occurred among related genomes at loci that otherwise seem collinear at in genetic mapping [65]. These results indicate that a closer look at gene-level collinearity is needed.

Single-pass BAC-end sequences are generally very specific and hence can be used as markers for comparative genomic studies. The BES reported here covers 16.4% of switchgrass genome and thus provides a reliable resource for anchoring switchgrass sequences to related grass model genomes. We picked four genomes with varying evolutionary distances viz., sorghum, maize, rice and *Brachypodium*, for genome-wide comparisons with switchgrass. Based on the BES mapping, we identified 3338, 2400 and 1568 putative microsyntenic regions with sorghum, rice and *Brachypodium*, respectively. Identification of orthologous segments in these regions may facilitate functional genomic studies in switchgrass.

Comparisons of full-length BAC sequences of switchgrass also revealed its higher similarity to sorghum followed closely by rice and then maize and *Brachypodium*. Sorghum and maize diverged from switchgrass about 28 million years ago [66]; whereas, rice and *Brachypodium* have diverged from switchgrass >50 and 60 mya, respectively. Reiterating the significance of genic-level sequence comparisons, the phylogenetic divergence between these genomes does not correspond to the pattern of collinearity we observed.

Due to difficulty of cloning and characterizing genes in polyploids like switchgrass; rice and *Brachypodium* have been promoted as surrogates for gene discovery and genomic analysis of other grasses [65]. Our results suggest that findings from the model genomes can be utilized for initiating functional genomic studies in switchgrass. However, due to widespread genome rearrangements, sorghum, along with soon to-be-completed foxtail millet genome will better serve as reference for assembling the genic region of the switchgrass genome.

It will be intriguing to investigate what makes switchgrass so different from these crops in terms of morphology, effective genome size (∼1600 Mbp; four times than that of rice), ploidy level (polyploid vs diploid rice) and physiological processes (C4 vs C3 in rice and *Brachypodium*). Certainly, the substantial rearrangements observed in some of the BACs would contribute to these factors. The set of genes identified from switchgrass that lack syntenic matches with other genomes may represent lineage-specific loci with novel or divergent functions. Detailed analysis of switchgrass gene functions is needed to enlighten this area.

The results reported here represent an important milestone for advancement of functional and comparative genomic studies of switchgrass. The BAC library resources and comparative anchoring of BES will be useful for SSR marker development, saturating existing linkage maps, anchoring physical and genetic maps, and assembly of ongoing genome sequence of switchgrass.

## Materials and Methods

### Plant Material and HMW DNA Preparation

Leaf tissue from young plantlets of *Panicum virgatum* L. cv. Alamo clone AP13, provided by the group of Michael Udvardi at The Samuel Roberts Noble Foundation, was used for preparation of high molecular weight (HMW) DNA. Briefly, nodes from greenhouse-grown plants were sterilized with 20% commercial bleach containing 0.1% Tween 20 followed by *in vitro* culture. New shoots were cut and transferred to rooting medium. Leaf tissue was harvested from plantlets after 16 h of dark treatment and frozen in liquid nitrogen.

### BAC Library Construction

BAC libraries were constructed at Clemson University Genomics Institute (CUGI) according to a published protocol [67] with minor modifications. Briefly, 100 g tissue was ground to powder in liquid nitrogen with pestle and mortar, and nuclei were isolated. To remove charged molecules as well as small and sheared gDNA, nuclei embedded into agarose plugs were exposed to pre-electrophoresis by loading onto a 1% TBE CHEF gel under the following conditions: 1 to 4 s switch times run at 4 V/cm for 3 h at 14°C. Genomic DNA was digested with *Hin*dIII and *Bst*Y1 restriction enzymes, separately, and large fragments were retrieved from gel fractions. *Hin*dIII and *Bst*YI digested fragments were used for DNA ligation into *Hin*dIII and *Bam*HI digested and dephosphorylated pIndigoBAC536 vectors [67], respectively.

### Gene Copy Number Estimation

For Southern blot analysis, total genomic DNA was isolated from leaf tissue of *Panicum virgatum* L. var. Alamo clone AP13 as described [68]. Briefly, 1 g frozen leaf tissue was ground to fine powder using a pre-chilled mortar and pestle. Powdered leaf tissue was transferred to a 30 mL centrifuge tube containing 15 mL extraction buffer (100 mM Tris-HCl, pH 8.0; 50 mM EDTA, pH 8.0; 500 mM NaCl and 10 mM β-mercaptoethanol). After lysis with 20% sodium dodecyl sulphate (SDS), DNA was precipitated using isopropanol and treated with RNase A (30 µL of 10 mg/mL stock per sample) for 1–2 h at 37°C. The samples were extracted once with phenol∶chloroform∶isoamyl alcohol, followed by another extraction with chloroform∶isoamyl alcohol only. DNA was precipitated with 0.1 volume of 3 M sodium acetate and 2.5 volumes of absolute ethanol for 1 h at −20°C.

Aliquots of genomic DNA (12 µg each) were digested with four different restriction enzymes (*Bam*HI, *Eco*RI, *Hin*dIII and *Sac*I) separately. Digested DNA samples were analyzed on 0.8% w/v agarose gel and blotted on nylon membrane (Hybond-N+™, Amersham Pharmacia Biotech Ltd.) by capillary transfer. To prepare probes, gene-specific primers were designed for known single copy genes from closely related genera (rice and maize) of the Poaceae family. The list of primers is given in File S10. DNA fragments, amplified using switchgrass DNA as a template, were labeled with alkaline phosphatase enzyme using Amersham Gene Images AlkPhos Direct Labeling and Detection System from GE Healthcare). Hybridizations and detection were performed according to manufacturer's instructions. In brief, approximately 5 ng probe was used per mL of hybridization buffer. Hybridizations of labeled DNA with membrane filters were performed overnight at 60°C in hybridization oven using hybridization bottles at 10 rpm. Primary washes were performed at 58°C for 20 min each. CDP-Star™ chemiluminescent detection reagent was used for signal generation. Chemiluminescence was captured on an X-ray film, purchased from ISC-BioExpress USA and recorded using a document scanner.

### Library Characterization

Approximately, 180 BAC clones were randomly selected from each library and inoculated to 2 mL overnight cultures of LB media containing 12.5 µg/mL chloramphenicol in 15 mL culture tubes. Cells were collected at 16,000 g for 10 min and BAC DNA was prepared using Qiagen's plasmid isolation kit. BAC DNA was digested with 10 U of *Not*I and analyzed on an agarose gel. Insert size of BAC clones was estimated by comparing with the Lambda ladder PFG marker (New England Biolabs Inc.) as standard. High-density filter hybridizations were performed to check extra-nuclear DNA contamination and library coverage. Each filter contained 18,432 individual clones, arrayed in a 4×4 pattern in duplicate. Gene-specific DNA sequences (500–1000 bp in length) spanning through chloroplast (*trn*L, *rpo*B, *ndh*A and *rbc*L) and mitochondria (*atp*6, *atp*9, *cob* and *cox*1) genomes of rice/sorghum were used to design primers (File S10). The corresponding DNA sequences were amplified using switchgrass genomic DNA, labeled and used for filter hybridizations, as described earlier. The Clarke-Carbon equation [69], N = ln(1−P)/ln(1−[I/GS]), where N is the number of clones, GS is genome size and I is insert size, was used to calculate the theoretical probability of finding a sequence of interest among the BAC clones.

### Full-length BAC Sequencing

Essentially full-length sequences for randomly selected BAC clones were obtained at the HudsonAlpha Institute of Biotechnology (www.hudsonalpha.org) by Sanger's method on ABI 3730XL DNA analyzers. The resulting trace data was base called using Phred V 0.020425. The Phred/Phrap/Consed suite of programs was used for assembling and editing the sequence [70], [71], [72]. After manual inspection of the assembled sequences, finishing was performed both by re-sequencing plasmid subclones and by primer walking on plasmid subclones or the BAC clone using custom primers. All finishing reactions were performed using dGTP BigDye Terminator Chemistry (Applied Biosystems). Hard-to-sequence gaps or small repeats were completed using small insert shatter libraries generated using Roche/454 sequencing technology or transposon libraries generated using Sanger technology.

### BAC-End Sequencing (BES)

The BES reads were obtained by Sanger's method on ABI 3730XL capillary sequencing machines at the HudsonAlpha Institute of Biotechnology. The resulting trace data was base called using Phred V 0.020425 and vector sequences were masked using cross_match. Masked terminal vector sequences and BES less than 50 bp in length were removed. High quality sequences were then filtered for plant-organelle genomes-specific or *Escherichia coli*-specific sequences.

### Analysis of Simple Sequence Repeats (SSRs) and Repeat Elements

We used mreps [73], a simple repeat identification software, to identify Simple Sequence Repeats (SSRs) from fasta-formatted unique BES. Parameters used were 1–3 nt repeats at least 12 nt in length and 4–6 nt repeats with at least 4 unit repetition. Other known repeat elements like TEs, rRNAs, centromere-/telomere-related sequences were identified with RepeatMasker 3.3.0 (http://www.repeatmasker.org/) [74], [75] and AB-BLAST v3 (http://blast.advbiocomp.com/) using the Viridiplantae section of the RepBase repeat database (release 20110419) [76]. To identify novel repeat elements, switchgrass BES were masked with RepeatMasker 3.3.0 [75] and compared to themselves using MegaBlast (E-value = 10^−50^). BES with at least six hits were analyzed using MEME V3.5.7 [77] to identify DNA motifs (E-value = 10^−4^). Resulting putative switchgrass repeat elements (SREs) were queried in the RepBase repeat database (release April 2011) [76], MSU Plant Repeat Database release May 2009 [78], Triticeae repetitive sequence database (TREP) (release 10; http://wheat.pw.usda.gov/ITMI/Repeats/index.shtml), NCBI GenBank non-redundant nucleic acid sequence database (Release 184.0; http://www.ncbi.nlm.nih.gov/RefSeq/) and Swissprot database (release August 2011; http://www.ebi.ac.uk/uniprot/) with BLASTN and BLASTX under E-value cutoff of 10^−4^ to check for their uniqueness.

### Functional Annotation and GO Analysis

Gene predictions from switchgrass BES was performed using Geneid v 1.4.4 [79] and PASA (http://pasa.sourceforge.net/). Predicted proteins were functionally annotated by comparison with Pfam database (version 25.0) using HMMER 3.0 [79]. GO terms were converted from Pfam domains using the mapping tool of the Gene Ontology project (http://www.geneontology.org/).

### Comparative Mapping of BAC-end Sequences

To map BAC-end sequences onto grass genomes, the BES were first aligned to rice peptide sequences using BlastX. The equivalent regions in sorghum and *Brachypodium* were identified and used for mapping BES. All genome sequences were extracted from Phytozome (http://www.phytozome.net/). Best alignments were identified for each BES that placed above a base pair identity of 75% with e value <1e-20, and coverage of the BES >50%. Furthermore, a best placement for BES that aligned to multiple locations after applying the aforementioned screening criteria was determined by sorting the placements using the blast score. Pairs were identified with a maximum insert size of 500 KB. If only one side of the pair placed in coding sequences, then we performed a blast alignment of the mate on the nucleotide sequence of the whole rice region (equivalently in *Brachypodium* and sorghum) to find the mate. The syntenic relationship among genomes and mapping results are displayed using the Gbrowse-syn module [21].

### Gene Annotations and Mapping of Full-length BAC Sequences

To produce high-quality non-redundant genomic sequences, repeat elements from full-length BAC sequences were masked using RepeatMasker 3.3.0 [75]. Gene models were identified using GenomeScan (http://genes.mit.edu/genomescan.html). Further PASA (http://pasa.sourceforge.net/) and NCBI EST sequences were used to update GenomeScan predictions. BLAST analysis to rice and *Arabidopsis* databases (http://rice.plantbiology.msu.edu/, http://www.arabidopsis.org/) and Pfam domain analysis (http://pfam.janelia.org/search) was performed to identify the conserved domains.

Genomic sequences of sorghum and *Brachypodium* were downloaded from Phytozome v6.0 (http://www.phytozome.net/), maize from MaizeSequence release 5b.60 (http://www.maizesequence.org) and of rice from MSU v6.1 (http://rice.plantbiology.msu.edu/). Discontinuous mega blast with a cutoff of 1e^−**20**^ was used to compare switchgrass gene models with other grass genomes. The microcolinearity among genomes was visually identified and displayed using Adobe Illustrator CS4. Direction of genes was determined using online databases (http://rice.plantbiology.msu.edu/; http://www.phytozome.net/).

The libraries and filters have been made available to the public through the Clemson University Genomics Institute (CUGI; www.genome.clemson.edu). Full-length BAC sequences for randomly selected 47 BAC clones have been submitted to GenBank under accession numbers AC243215–AC243261. GenBank accession numbers for BES are HR309496–HR503629 (Pv_ABa) and JM786703–JM972700 (Pv_ABb).

## Supporting Information

Figure S1 **Southern hybridizations for gene copy number estimations in switchgrass.** We used Southern hybridizations to determine the copy number, of single/low copy genes from closely related monocotyledonous plant species in switchgrass. The results of Southern hybridizations using four different restriction enzymes for each gene are presented.
(JPG)

File S1 **Distribution of simple sequence repeats identified in switchgrass BAC-end sequences.**
(XLS)

File S2 **List of nucleotide sequence of novel switchgrass repetitive repeats (SREs).**
(TXT)

File S3 **Distribution of GO annotations with regard to A, Functional classes of gene products encoded from BAC end sequences; B, Biological processes associated with gene products and their C, cellular locations.**
(XLS)

File S4 **Distribution of simple sequence repeats and plant repeat elements identified from full-length BAC sequences.**
(DOC)

File S5 **List of 439 switchgrass gene loci (451 gene models) annotated from switchgrass full-length BAC sequences.**
(XLS)

File S6 **List of A, cDNA; B, genomic and C, protein sequences of switchgrass genes predicted from full-length BAC sequences.**
(XLS)

File S7 **List of switchgrass gene models with their corresponding orthologs from rice, sorghum, maize and** ***Brachypodium*** **.**
(XLS)

File S8 **List of genes from rice, sorghum, maize and** ***Brachypodium*** **that are not present in the corresponding regions in switchgrass in** **figure 8** **.**
(XLSX)

File S9 **Genome characteristics of various plant genomes based upon BAC-end or genome sequence data.**
(XLS)

File S10 **List of primers used for BAC library characterization and Southern hybridizations.**
(DOC)
