# Supplementary figures and images for: A Genome-Wide Survey of Switchgrass Genome Structure and Organization
Source: PLoS One. 2012 Apr 12;7(4):e33892. doi: 10.1371/journal.pone.0033892 (PMC3325252; doi:10.1371/journal.pone.0033892)

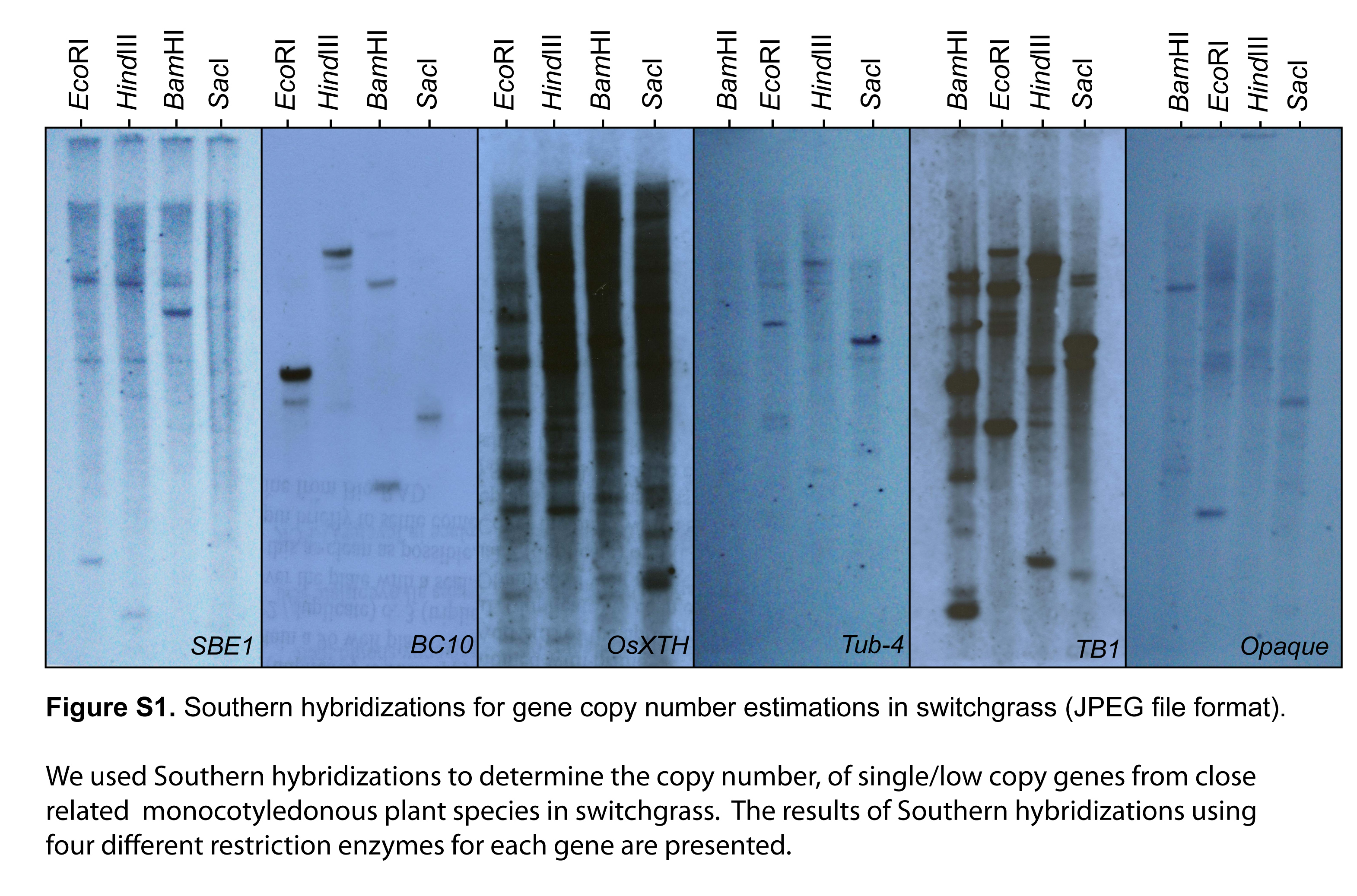

Supplement: Figure S1 — Southern hybridizations for gene copy number estimations in switchgrass. We used Southern hybridizations to determine the copy number, of single/low copy genes from closely related monocotyledonous plant species in switchgrass. The results of Southern hybridizations using four different restriction enzymes for each gene are presented. (JPG) [file pone.0033892.s001.jpg]
