# Supplementary material for: A Genome-Wide Survey of Switchgrass Genome Structure and Organization
Source: PLoS One. 2012 Apr 12;7(4):e33892. doi: 10.1371/journal.pone.0033892 (PMC3325252; doi:10.1371/journal.pone.0033892)
Supplement: File S4 — Distribution of simple sequence repeats and plant repeat elements identified from full-length BAC sequences. (DOC) [file pone.0033892.s005.doc]

**Supplemental file S4.** Distribution of simple sequence repeats and plant repeat elements identified from full-length BAC sequences.

**Simple Sequence Repeats**

In parallel to BES analysis, we also analyzed randomly selected full-length BAC clones (47 in number) for estimation of simple sequence repeats (SSRs) and repeat content. We found 1341 SSRs from 7.2 Mbp of full-length BAC sequences. Therefore, SSR density in BES and full-length BACs is similar. As shown with BES, trinucleotide repeats were most abundant (47.88%), followed by dinucleotides (27.89%) and mononucleotide (18.27%) repeats. CCG/CGG was the most common trinucleotide repeat, whereas, C/G seems the most abundant mononucleotide repeat.

| **Distribution of SSRs identified from full-length BAC sequences.** | | |
| --- | --- | --- |
| **Type** | **Sequence** | **Number** |
| **Monomer** | A/T | 68 |
|  | C/G | 177 |
| **Dimer** | AC/GT | 85 |
|  | AG/CT | 152 |
|  | AT | 126 |
|  | CG | 11 |
| **Trimer** | AAC/GTT | 25 |
|  | AAG/CTT | 88 |
|  | AAT/ATT | 25 |
|  | ACC/GGT | 43 |
|  | ACG/CGT | 31 |
|  | ACT/AGT | 3 |
|  | AGC/GCT | 67 |
|  | AGG/CCT | 95 |
|  | ATC/GAT | 24 |
|  | CCG/CGG | 241 |
| **Tetramer** | AAAC/GTTT | 1 |
|  | AAAG/CTTT | 7 |
|  | AAAT/ATTT | 4 |
|  | AAGC/GCTT | 4 |
|  | AATC/GATT | 1 |
|  | AATG/CATT | 1 |
|  | AATT | 1 |
|  | ACAT/ATGT | 5 |
|  | ACCT/AGGT | 1 |
|  | ACGC/GCGT | 1 |
|  | ACGG/CCGT | 1 |
|  | ACTG/CAGT | 1 |
|  | AGCC/GGCT | 1 |
|  | AGCG/CGCT | 1 |
|  | AGGC/GCCT | 1 |
|  | AGGG/CCCT | 6 |
|  | ATCC/GGAT | 4 |
|  | ATCG/CGAT | 2 |
|  | ATGC/GCAT | 2 |
|  | CCCG/CGGG | 3 |
|  | CCGG | 3 |
| **Pentamer** | AAAAC/GTTTT | 1 |
|  | AAAAG/CTTTT | 2 |
|  | AAATG/CATTT | 1 |
|  | AAGAG/CTCTT | 1 |
|  | AATAT/ATATT | 1 |
|  | ACAGT/ACTGT | 1 |
|  | ACCGC/GCGGT | 3 |
|  | ACGAG/CTCGT | 1 |
|  | ACGCG/CGCGT | 1 |
|  | AGCCG/CGGCT | 2 |
|  | ATCCC/GGGAT | 1 |
|  | CCCGG/CCGGG | 1 |
|  | CCGCG/CGCGG | 4 |
| **Hexamer** | AACAGC/GCTGTT | 1 |
|  | AATACT/AGTATT | 1 |
|  | AATGGG/CCCATT | 1 |
|  | ACATAT/ATATGT | 1 |
|  | ACCTGC/GCAGGT | 1 |
|  | ACGGCC/GGCCGT | 1 |
|  | AGAGGC/GCCTCT | 2 |
|  | CCCCCG/CGGGGG | 1 |
| Total number of SSRs - 1341 | | |
| Total length of nucleotide sequence - 21810 bp | | |
|  | | |
| **Length distribution** | |  |
|  | 12-20nt | 1186 |
|  | 21-50nt | 118 |
|  | 51-100nt | 36 |
|  | >100nt | 1 |

**Repeat Elements**

Analysis of repetitive elements revealed 3429 sequences (Supplemental Table S9) showing significant homology with plant repeat elements that represent 25.47% of total sequence analyzed. 1107 low complexity regions were also identified. Similar to repeat elements in BES, transposable elements (TEs) predominate and constitute a significant fraction (25.14%) of sequence analyzed.

| **Distribution of plant repeat elements identified from full-length BAC sequences.** | | | |
| --- | --- | --- | --- |
| **Category** | **Number of Elements** | **Sequence occupied (bp)** | **% of total sequence analyzed** |
| **Retrotransposones** | 1714 | 1386164 | 19.2 |
| SINEs | 49 | 8649 | 0.12 |
| LINEs | 150 | 97610 | 1.35 |
| *RTE/Bov-B* | 34 | 26310 | 0.36 |
| *L1/CIN4* | 116 | 71300 | 0.99 |
| LTR Retroelements | 1518 | 1279905 | 17.73 |
| *Ty1/Copia* | 453 | 457411 | 6.34 |
| *Gypsy/DIRS1* | 1035 | 816533 | 11.31 |
| **DNA Transposons** | 1601 | 434005 | 6.01 |
| Hobo-Activator | 168 | 43822 | 0.61 |
| Tc1-IS630-Pogo | 123 | 20373 | 0.28 |
| En-Spm | 561 | 192320 | 2.66 |
| MuDR-IS905 | 293 | 88810 | 1.23 |
| Tourist/Harbinger | 356 | 72874 | 1.01 |
| **Total Transposable Elements** | **3315** | 1820169 | 25.21 |
|  |  |  |  |
| **Unclassified** | **114** | 18125 | 0.25 |
|  |  |  |  |
| Total Interspersed repeats |  | 1838294 | 25.47 |
| Small RNA | **2** | 3342 | 0.05 |
| Satellites | **5** | 294 | 0.01 |
| Low complexity | **1107** | 52405 | 0.73 |
