# Supplementary material for: A Genome-Wide Survey of Switchgrass Genome Structure and Organization
Source: PLoS One. 2012 Apr 12;7(4):e33892. doi: 10.1371/journal.pone.0033892 (PMC3325252; doi:10.1371/journal.pone.0033892)
Supplement: File S10 — List of primers used for BAC library characterization and Southern hybridizations. (DOC) [file pone.0033892.s011.doc]

**Supplemental table S10**. List of primers used for BAC library characterization and Southern hybridizations.

|  | **Name** | **Primer** | **Sequence** |
| --- | --- | --- | --- |
| Chloroplast-specific genes | *rbcL* | For | 5′-TACTTCCATTGTGGGTAACGTAT-3′ |
|  |  | Rev | 5′-AGATACCGCGAGAACGATCT-3′ |
|  | *ndhA* | For | 5′-TAACTTTTTGTGTACTAGCAATATCTC-3′ |
|  |  | Rev | 5′-CTGTGCTTCAACTATATCAACTGT-3′ |
|  | *rpoB* | For | 5′-ATATGGGCTCGCGTGAGT-3′ |
|  |  | Rev | 5′-GACAAATACGCCCATAGTGA-3′ |
|  | *trnL* | For | 5′-GGACTTGATATGTATTGAGCCT-3′ |
|  |  | Rev | 5′-TAAAGTCGACGGATTTTCCT-3′ |
| Mitochondria-specific genes | *atp6* | For | 5′-CAAATGCATGGCAATCCTT-3′ |
|  |  | Rev | 5′-TGAGCTTGTGATATAGCTACACCT-3′ |
|  | *atp9* | For | 5′-AGGAAAAGCGCGAAACAAT-3′ |
|  |  | Rev | 5′-GACCAATGTGTATACGCACCA-3′ |
|  | *cob* | For | 5′-AGCGCCATACCAGTAGTAGGA-3′ |
|  |  | Rev | 5′-ATGGGCGTTATGGCAAAGA-3′ |
|  | *cox1* | For | 5′-TCTATGCATAGTCATTCCAGGT-3′ |
|  |  | Rev | 5′-CTAGTTTGAAGTTCTCCTTGAGT-3′ |
| Single or low copy genes | *BC10* | For | 5′-TGCACCAACCTAGGTACTAAAAGA-3′ |
|  |  | Rev | 5′-ACTATGTTCAGACATTGCTTGCA-3′ |
|  | *Os_XTH* | For | 5′-CGGACTCGTGGCTGTAC-3′ |
|  |  | Rev | 5′-ACTTTCACCTTCCCCTGCT-3′ |
|  | *Maize opaque* | For | 5′-AGAGAATCAGCCAGACGCT-3′ |
|  |  | Rev | 5′-GCAGTGCCTAATACATGTCCA-3′ |
|  | *SBE1* | For | 5′-ACTCACACGCCGTCACAAT-3′ |
|  |  | Rev | 5′-TTGGGGTCCAGGTCGTATA-3′ |
|  | *Tubulin-4* | For | 5′-ACAACTTCGTCTACGGCCAGT-3′ |
|  |  | Rev | 5′-CTTGGCGTCCCACATCTGCT-3′ |
|  | *TB1* | For | 5′-TCTCACGCTCACGCTCACA-3′ |
|  |  | Rev | 5′-ACGCGTCGTCTGTCATGATCT-3′ |
